# Perceptions of interprofessional collaboration of general practitioners and community pharmacists - a qualitative study

**DOI:** 10.1186/s12913-017-2157-8

**Published:** 2017-03-21

**Authors:** Christin Löffler, Carolin Koudmani, Femke Böhmer, Susanne D. Paschka, Jennifer Höck, Eva Drewelow, Martin Stremme, Bernd Stahlhacke, Attila Altiner

**Affiliations:** 1 Institute of General Practice, Rostock University Medical Center, Doberaner Str. 142, 18057 Rostock, Germany; 2 Department of Conservative Dentistry and Periodontology, Rostock University Medical Center, Strempelstr. 13, 18057 Rostock, Germany; 3 Hospital Pharmacy, Rostock University Medical Center, Ernst-Heydemann Str. 7, 18057 Rostock, Germany; 4 Chamber of Pharmacists of Mecklenburg-Western Pomerania, Wismarsche Str. 304, 19055 Schwerin, Germany

**Keywords:** Interprofessional relations, Pharmacists, General practitioners, Primary health care, Polypharmacy, Qualitative research

## Abstract

**Background:**

Despite numerous evidences for the positive effect of community pharmacists on health care, interprofessional collaboration of pharmacists and general practitioners is very often limited. Though highly trained, pharmacists remain an underutilised resource in primary health care in most western countries. This qualitative study aims at investigating pharmacists’ and general practitioners’ views on barriers to interprofessional collaboration in the German health care system.

**Methods:**

A total of 13 narrative in-depth interviews, and two focus group discussions with 12 pharmacists and general practitioners in Mecklenburg-Western Pomerania, a predominantly rural region of North-Eastern Germany, were conducted. The interviews aimed at exploring general practitioners’ and pharmacists’ attitudes, views and experiences of interprofessional collaboration. At a second stage, two focus group discussions were performed. Fieldwork was carried out by a multi-professional team. All interviews and focus group discussions were audio taped and transcribed verbatim. The constant comparative method of analysis from grounded theory was applied to the data.

**Results:**

There are three main findings: First, mutual trust and appreciation appear to be important factors influencing the quality of interprofessional collaboration. Second, in light of negative personal experiences, pharmacists call for a predefined, clear and straightforward way to communicate with physicians. Third, given the increasing challenge to treat a rising number of elderly patients with chronic conditions, general practitioners desire competent support of experienced pharmacists.

**Conclusions:**

On the ground of methodological triangulation the findings of this study go beyond previous investigations and are able to provide specific recommendations for future interprofessional collaboration. First, interventions and initiatives should focus on increasing trust, e.g. by implementing multi-professional local quality circles. Second, governments and health authorities in most countries have been and still are reluctant in advancing political initiatives that bring together physicians and pharmacists. Proactive lobbying and empowerment of pharmacists are extremely important in this context. In addition, future physician and pharmaceutical training curricula should focus on comprehensive pharmacist-physician interaction at early stages within both professional educations and careers. Developing and fostering a culture of continued professional exchange and appreciation is one major challenge of future policy and research.

## Background

Along with demographic and epidemiological changes a growing number of patients with multiple chronic diseases need to be cared for. In primary care, general practitioners (GPs) across Europe, the US and Australia are increasingly supported by other qualified health professions, such as nurse practitioners or practice nurses. Interprofessional collaboration with community pharmacists, though, did not develop to the same extent. In general, GP-pharmacist interactions are of low frequency [1–3].

This is striking since numerous studies from various settings provide evidence for the positive effect of community pharmacists on medication management, patient counselling, health education, and improved care resulting in better clinical outcomes [4–6]. A recent Cochrane Review shows that pharmacist interventions are particularly beneficial for reducing systolic blood pressure, improving HbA1C and blood glucose level, and for the management of asthma [4].

However, interprofessional collaboration of pharmacists and GPs is very often limited to the clarification of inaccurate prescriptions [3, 7] or the provision of drug-related information [8]. For instance, Urban and colleagues found that the potential of community pharmacists to improve patient safety after discharge from hospital is not being used in England [9]. Across Europe pharmacists are infrequently involved in patient-centred professional activities such as monitoring plans [10]. The situation in the US differs to some extent as the implementation of collaborative working relationships between community pharmacists and physicians aims at fostering interprofessional collaboration. Yet collaboration is often limited by compensation barriers and a lack of initiative [11]. Though highly trained, in most western countries pharmacists are in fact an underutilised resource within the primary health care sector [12].

First attempts to explain this development point to GPs’ concerns about an increasing workload and patient confusion, as well as a different perception and importance assigned to trust and communication [13, 14]. Ambler suspects that existing barriers are highly related to the fact that GPs and pharmacists do not know one another very well [1]. In fact, a comprehensive understanding of the way GPs and community pharmacists perceive the others profession, is still missing. The aim of this paper was to investigate the views of pharmacists and GPs on barriers to interprofessional collaboration in the German health care system.

## Methods

### Design and setting

This work is part of a pilot study that has been conducted at Rostock University Medical Center, Germany. Whereas the pilot study aimed at developing and testing a focused localized intervention, this work pursued a wider aim by addressing existing barriers to interprofessional collaboration between GPs and pharmacists in the German health care system. In Germany, most pharmacies are small-sized private enterprises mainly concerned with drug disposal. Pharmacists usually provide basic drug information; they are rarely involved in drug management. There is no public drug program providing medication reviews or similar services.

In 2012/2013 we conducted narrative in-depth interviews and focus group discussions with community pharmacists and GPs in Mecklenburg-Western Pomerania, a predominantly rural region of North-eastern Germany. The narrative interviews were carried out by CK and FB (both young female physicians). Interviewers and interviewees have not met each other before the interview. First, seven GPs and six community pharmacists were interviewed. The interviews aimed at exploring GPs’ and pharmacists’ attitudes, views and experiences of interprofessional collaboration. Narrative interviews have been developed in the 1970s to stimulate memory and narration. They aim at motivating interviewees to narrate extensively about self-experienced events and to reconstruct courses of action and ex-post evaluations [15]. Narrative interviews are characterized by an initial phase of topic formulation, which is carried out by the interviewer. The initial phase is followed by the main narrative phase of the interviewee, where the interviewer only takes notes. When the interviewee stops talking, the interviewer refers to the information gained so far and stimulates the interviewee to narrate and describe further details. Then external open-ended questions, formulated prior to the interview, are asked [16]. External questions addressed to the pharmacists included, for instance, positive and negative experiences and situations of interprofessional collaboration with GPs. Equally GPs were asked to report their experiences with regard to collaborations with pharmacists. Subsequent to each interview, the interviewer drafted a memo to summarize the interview situation, the information gained and first ideas for the analyses.

At a second stage, emerged perceptions, concepts and potential solutions that arose from the interviews provided the basis for possible localized interventions and were reflected in the group discussions [17]. The major purpose of the focus group discussions was to debate on feasibility and acceptability of those potential interventional concepts to improve interprofessional collaboration. Once having completed interviews, we performed two focus group discussions with a total of eight GPs and four pharmacists. As the interviews already provided insight into the potential for conflict between both professions, the first focus group discussion was performed with GPs only. Four GPs participated in this discussion. Potential interventional concepts were modified after that discussion. Another four GPs and four pharmacists took part in the second discussion. A team of two researchers, BS and CL (a male pharmacist and a female sociologist) moderated the discussions and encouraged participants to share their perceptions and point of views, e.g. on advantages and disadvantages of different paths of communication. The questions raised were based on previous findings from interviews. The interviews had an average length of 30 min, the focus group discussions lasted about 90 min each. Interviews were stopped as soon as additional data collection did not provide any new insights. During the focus group discussions, participants extensively discussed and refined the interventional elements that emerged from the interviews. They also agreed on potential procedures for interprofessional collaboration, which were tested in a pilot study. This paper focuses on pharmacists’ and GPs’ views on barriers to interprofessional collaboration. It is not the intention of this work to provide respondents evaluation of the proposed interventions, which will be published elsewhere.

### Interviewee recruitment and sample description

Community pharmacists were selected from a contact list of local community pharmacists provided by the Chamber of Pharmacists of Mecklenburg-Western Pomerania. A total of 15 pharmacists, who participated in a previous study on polypharmacy among elderly, were contacted via mail. Ten of them agreed to take part. Six pharmacists were interviewed and four pharmacists took part in the focus group discussion. GPs were recruited through the local teaching and research network of the Institute of General Practice at the Rostock University Medical Center. 40 GPs were randomly contacted by mail from which 15 were interested. Seven GPs were interviewed and eight GPs participated in the focus group discussions. Thus, in total 25 interviewees participated in this qualitative study. Two of the pharmacists were male and eight were female; age ranged from 31 to 44 years. Among GPs eight were male and seven female; age ranged from 37 to 75 years. Interviewees of both professions worked in places with different levels of urbanisation, ranging from rural to medium-sized and urban.

### Data analyses

All interviews and focus group discussions were audio taped and transcribed verbatim. Since the authors of this paper belong to various disciplines, the analyses benefitted from a multidisciplinary perspective: Additionally to memos drafted right after the interviews and focus group discussions, the authors drafted thematic memos and discussed them regularly. We applied the constant comparative method of analysis from *grounded theory* [18–20] to the data by employing the three steps of coding and categorizing: *open coding*, *axial coding,* and *selective coding.* Coding and categorising of the interviews was done by CK and CL and discussed continuously with the other researchers through the entire process of analysing. This proceeding also increased coding reliability. All data was managed and coded by using a qualitative data software program (*QSR NVivo version 9*).

### Presentation of findings

**Fig. 1:**
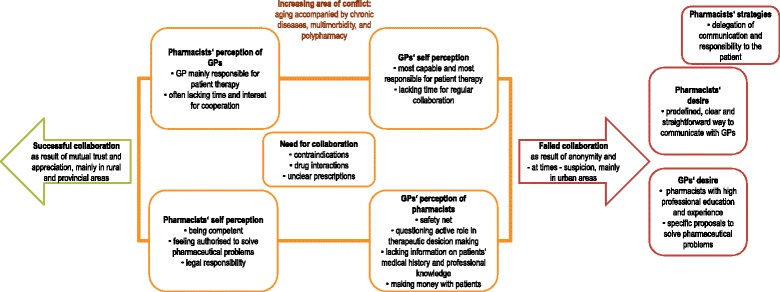
Categories influencing GPs’ and pharmacists’ views on interprofessional collaboration

A number of categories turned out to influence pharmacists’ and GPs’ views on interprofessional collaboration. These categories arose from both, the interviews and the focus group discussions. As they are highly interwoven with each other, findings are organized in a way to contrast pharmacists’ with GPs’ points of view and experiences with interprofessional collaboration. Relevant categories are summarized and presented in Fig. 1. The quotes used to illustrate our results were translated by a professional bilingual translator. Information that would allow indirect identification of interviewees or focus group participants was removed.

## Results

### Interprofessional collaboration from the pharmacists’ point of view

The qualitative data material showed that the interviewed community pharmacists felt competent and authorised to solve pharmaceutical problems. They emphasised their legal responsibility to take care of the medication provided to patients. In doing so they wished for a stronger support by the GPs. Almost all participating pharmacists saw a need to improve the doctor-pharmacist dialog.

Community pharmacists reported that major occasions requiring contact to GPs include contraindications, drug interactions, and unclear prescriptions. Whereas some of the participating pharmacists made, in general, positive experiences when contacting GPs, the majority stated to encounter recurring difficulties getting GPs on the phone and receiving an answer to their query. Rather than perceiving these queries as supportive, pharmacists often had the feeling that GPs considered requests as invasive and controlling. Further obstacles mentioned were physicians’ time constraints and the non-cooperation of nurses in connecting pharmacists and physicians on phone. Several pharmacists reported about negative experiences, malicious insults and a lack of respect when contacting physicians. One female pharmacist talked about the effect negative reactions of physicians have on her work. When fearing verbal abuses she usually avoids calling physicians and tries to raise patients’ awareness of potential pharmaceutical problems:

*“Sometimes we actually fear calling there, because we are scared of being snapped at. You know, we’ve sometimes had such bad experiences. Of course, we’ve also had really good experiences, but you know, if you have ten really good experiences and only one bad one it is the bad experience that sticks. […] So that is when you get scared and say, “are you really going to call him?” […] And if I end up telling myself, “God no, I’m not even going to call him!”, then I try to somehow sensitize the patient – at least a little, right? Or you end up telling them, “why don’t you go back there and ask him yourself one more time?”*

Compared to pharmacists working in urban areas, those employed in rural and provincial regions reported less negative experiences when contacting GPs. They often experienced long-lasting working relationships to local physicians that were mostly characterized by mutual trust and appreciation. With this foundation interprofessional collaboration was more successful. In contrast, in cities interprofessional collaboration was constrained by urban anonymity: Quite often, pharmacists hardly knew the physicians they tried to contact. One pharmacist described the situation when contacting unknown physicians:

*“And of course, sometimes it’s those other doctors (GPs) whose offices are located a bit further away and whom you’re not constantly in touch with. That makes it difficult to reach them or to carefully explain something to them without giving them the feeling that, well, how should I put it, you’re stepping on their toes […]All that is always kind of tricky, that is, if you can reach them at all.”*

Being aware that, ultimately, physicians take decisions of patient medication, most pharmacists expressed the desire for a predefined, clear and straightforward way to communicate with physicians. They argued that a straightforward communication would avoid wrong assumptions and misunderstandings while aligning outcome-oriented physician-pharmacist communication.

### Interprofessional collaboration from GPs’ point of view

When asked about interprofessional collaboration with pharmacists, *initially* most GPs did not report any difficulties or obstacles. Some GPs - again primarily those from rural and medium-sized areas - emphasised their good relationships with local pharmacies. Most GPs reported about occasional phone calls from community pharmacies in case of unclear prescriptions. GPs generally appreciated this kind of safety net. However, only few GPs stated to contact pharmacists on their own initiative. Major reason given was the lack of time.

Not until delving into the focus group discussions, GPs questioned pharmacists’ active involvement into therapeutic decision-making. GPs perceived themselves as most capable and most responsible for patients’ medication. They felt that pharmacists miss background information on patients’ medical history and/or professional knowledge to understand and reconstruct physicians’ reasoning in many cases:

*“And when there is something I’m not sure of and I think to myself, “well, there used to be something, what was it called again?”, then of course I call! But that’s not the usual case, because pharmacists - well they simply are not trained that way. Well, at least not in terms of therapy. They are trained how to roll a pill aren’t they? I can check the effects and the other information right here on my own. I can assess interactions, contraindications and all that with the software I use. Those things have become a lot simpler due to the software.”*

In line with this, GPs clearly stated to appreciate pharmaceutical involvement when it was clinically relevant for the treatment of the patient. They emphasised that only high professional education and experience qualifies community pharmacists to decide whether certain information would be relevant for GPs or not. The benefit of receiving phone calls from “young, inexperienced pharmacists pointing to *relative* contraindications” was questioned by interviewed GPs. They underlined their desire for specific proposals to solve a given pharmaceutical problem. During one focus group discussion a GP stated, for instance, the following:

*“[That is why] it is so very important [to know] who is calling. Is it someone [clinically] experienced or somebody who is just calling due to a “bing” on his computer screen – those people tend to call the GP, too. It does, of course, play a role who approaches you. […] What I mean is, that we should immediately find a solution [in regard to the problematic prescription]. And then, when we do, to also find some kind of practical way to use that information.”*

In the same vein, another GP reported to feel stressed by those pharmacists just calling him without boiling information down to the essence. He finally enforced the rule that merely two experienced pharmacists of this pharmacy were allowed to contact him:

*“At the pharmacy next door there are two pharmacists, when they call, it instantly sets my teeth on edge. When they start rambling I always tell them “make it short, I have no time”. This of course leaves them dumbfounded, trying to remember what they were actually calling about and eventually they hang up because they just can’t stay focussed. (…) I simply don’t have the time to listen to some pharmacist’s gibberish. And I have to admit that I only know pharmacists who have plenty of time – in contrast to us. […] I have now established that while the chief pharmacist and maybe even the new colleague may call me, the others simply aren’t allowed to call me at all; because I know they’ll end up yapping forever without being able to make it short.”*

In light of a rising number of patients with chronic diseases and multimorbidity, time pressure was a major topic in the interviews and focus group discussions. GPs felt challenged in means of treating these patients under time constraints and avoiding or limiting polypharmacy. Most physicians perceived that community pharmacists were not able to respond to this challenge. Quite the contrary, some GPs thought that pharmacists would have their own agenda by making money with exactly this group of patients:

*“The problem is that pharmacies are promoting exactly this [the medicalization]. They sell blood-glucose-meters and, if they could, they would sell cholesterol testing devices and all kind of other stuff. It is them making people nervous. They even perform some kind of pseudo-venography. Osteoporosis-assessment, cholesterol-measurement, all of that just fuels the fire. While they want to sell more medication, we [the GPs] share a common interest to use less. At least I see it that way, and that’s just one more issue at hand, you know, that we have diverging interests.”*

## Discussion

By employing in-depth interviews and focus group discussions this qualitative study explores how German GPs and community pharmacists perceive both their interprofessional collaboration as well as the profession of the other.

There are three principal findings: First, mutual trust and appreciation - most prevalent among GPs and pharmacists from rural and medium-sized locations - appear to be important factors influencing the quality of interprofessional collaboration. There is evidence that anonymity strongly hampers the interaction between GP and pharmacist, whereas grown trust facilitates collaboration. Second, in light of negative personal experiences, pharmacists call for a predefined, clear, and straightforward way to communicate with physicians. In their view, such a communication frame would be able to strengthen interprofessional collaboration. This is also important with respect to GPs lack of time. Third, with regard to the rising number of elderly patients with chronic conditions, GPs desire competent straightforward support of experienced pharmacists. In light of the current pharmaceutical education and training also experts have emphasised that most German pharmacists lack clinical knowledge and experiences [21]. Actually, GPs perceived that pharmacists would have divergent interests by making money, especially with elderly patients suffering from chronic diseases.

The qualitative data provides evidence that based on negative experiences some community pharmacists avoid interprofessional contact and delegate responsibility for patient safety to the patient him-/herself. Patients, however, might not be able to evaluate the seriousness of the information gained. Non-communication might result in avoidable (serious) adverse events.

A previous survey conducted in Eastern Germany and investigating perceptions of pharmacists and physicians found that there is a foundation for a functional relationship between both professions in this setting [3]. Nonetheless, collaboration is of low frequency. Our study is the first that provides in-depth insights into existing barriers that are responsible for a low level of interprofessional interaction in this area. Focusing on different settings, some other qualitative studies analysed collaboration of community pharmacists and physicians. In New Zealand, Hatah and colleagues found that GPs fear patient confusion and increasing workload when involving pharmacists into patient counselling [13]. Though mentioned, these aspects were of minor importance in our setting. Bradley and colleagues concluded for England that factors such as trust, communication, professional respect and “knowing” each other were key components of collaboration [14]. This is in line with our findings from North-Eastern Germany.

Actually, the model of collaborative working relationships (CWR) that is used as theoretical framework in a number of quantitative studies investigating physician-pharmacist collaboration assumes that there are five progressive stages of collaboration: Starting with professional awareness progressing to professional recognition, exploration and trial, professional relationship expansion and finally commitment [22]. According to a survey with 239 Iowa pharmacists conducted by Liu and Doucette pharmacists need to show their skills and knowledge to physicians and clarify each party’s responsibilities in the care process in order to start collaboration [23]. Van et al. [24] also underlined the importance of early contact and proximity.

Most qualitative studies addressing physician-pharmacist collaboration make use of qualitative interviewing exclusively [2, 13, 14]. Focus group discussions or the triangulation of qualitative methods are rarely used [12]. The analyses of narrative in-depth interviews and focus group discussions in this study provide evidence, that the triangulation of methods is highly desirable when investigating this topic. Only during the focus group discussions, GPs and pharmacists started a dynamic discussion on experiences and provided numerous examples of failed collaboration. Qualitative interviews only, would not have provided these in-depth insights. The findings of this study go, in fact, beyond previous investigations and are able to provide specific recommendations for future interprofessional collaboration. Alongside with triangulation of qualitative methods, our study benefitted from a multidisciplinary perspective including several scientific backgrounds and professions. Nonetheless, the hypotheses generated in the context of rural Mecklenburg-Western Pomerania, do not necessarily have to be valid for other settings. In addition, confirmatory studies, such as a representative cross-sectional study, or a pilot study addressing current barriers, are necessary to further validate findings.

However, our findings have implications for policy makers and stakeholders. Existing barriers for pharmacist-physician interaction include a low level of mutual trust and appreciation, a missing framework for interprofessional communication, and the discordance about the importance of different pharmaceutical information. To improve interprofessional collaboration, firstly, interventions and initiatives should focus on increasing trust and appreciation. Obviously, doing so is much more difficult in urban areas than in rural regions. Local quality circles (periodic meetings of GPs) might be a good occasion to bring together both professions. Niquille and colleagues showed that in Switzerland physician-pharmacist quality circles were able to increase collaboration. Interestingly, compared to a control group among these circles drug costs were reduced by 42% [25]. Also, campaigns supporting pharmacists to introduce themselves and their pharmacies to local GPs might be discussed. Secondly, in most countries governments and health authorities have been and still are reluctant in advancing political initiatives that join physicians and pharmacists. Proactive lobbying [26] and empowerment of pharmacists are extremely important. Meanwhile, stakeholders should elaborate and provide recommendations for effective communication of pharmacists with physicians. Last, but not least, future physician and pharmaceutical-training curricula should focus on comprehensive pharmacist-physician interaction. Gallagher and Gallagher called for inter-professional and multi-professional learning opportunities at early stages within both professional educations. Different models have been developed in Scotland and the UK [26]. Developing and fostering a culture of continued professional exchange and appreciation is one major challenge of future policy and research.

## Conclusion

This qualitative study explores the perception GPs and community pharmacists have of their own profession as well as their perception of interprofessional collaboration. The data shows that there are three main barriers hampering physician-pharmacist interaction: First, a lack of mutual trust and appreciation, second an insufficiency of pre-defined communication structures and third, discordance about the importance of pharmaceutical information for patient care and treatment. Future initiatives and research should focus on developing, advancing and evaluating approaches to overcome these barriers and to increase interprofessional collaboration.

## Abbreviations

CWR: Collaborative working relationships
GP: General practitioners
